# Mechanical testing of transtibial prosthetic sockets: A discussion paper from the American Orthotic and Prosthetic Association Socket Guidance Workgroup

**DOI:** 10.1097/PXR.0000000000000222

**Published:** 2023-02-09

**Authors:** Francesca Gariboldi, Andrea Giovanni Cutti, Stefania Fatone, Eric Nickel, Alex Dickinson, Joshua Steer, Jeffrey Erenstone, Saeed Zahedi

**Affiliations:** 1 Department of Industrial Engineering, University of Padua, Padua (PD), Italy; 2 INAIL Prosthetic Center, Vigorso di Budrio (BO), Italy; 3 Department of Rehabilitation Medicine, University of Washington, Seattle, WA, USA; 4 Minneapolis VA Health Care System, US Department of Veterans Affairs, Minneapolis, MN, USA; 5 Faculty of Engineering & Physical Science, University of Southampton, Southampton, United Kingdom; 6 Radii Devices Ltd, Bristol, United Kingdom; 7 Performance Orthopedic Design, LLC, Lake Placid, NY, USA; 8 Blatchford, Basingstoke, United Kingdom

**Keywords:** prosthetic socket, testing, lower limb

## Abstract

The advent of novel manufacturing technologies, materials, and socket design concepts could introduce risks to prosthetic limb users, as the existing knowledge base for safe fabrication may not apply. Moreover, although structural test standards exist for mass-produced prosthetic components, they are not applicable to prosthetic sockets.

**Methods:**

The “AOPA Socket Guidance Workgroup” was formed in 2020 to provide the prosthetic community with evidence-based clinical best practices and methods in the field of prosthetic socket structural analysis. This multidisciplinary expert workgroup undertook a critical analysis of the knowledge gaps regarding the requirements for mechanical testing of lower limb prosthetic sockets.

**Results:**

The Workgroup identified knowledge gaps in 4 domains. Domain 1 describes the shape and composition of a mock residual limb, required to support and generate *in vivo* representative loading within the socket. Domain 2 concerns prosthetic socket coordinate systems and alignment. Domain 3 regards the components and requirements of test specimens. Finally, Domain 4 considers test conditions, loading parameters, and acceptance criteria.

**Conclusions:**

This paper describes these knowledge gaps in detail and recommends potential solution approaches based on literature review, group consensus around existing knowledge, or the formation of new study groups to fill each knowledge gap. Our intent is for the recommendations arising from this paper to support the community (e.g., researchers in the clinic, academia, industry, and funders) in addressing these knowledge gaps.

## Introduction

Lower limb prosthetic sockets serve as the personalized interface between the mass-produced, “engineered” components of the prosthesis (foot/ankle, structural connectors, and knee, if needed) and the residual limb, possibly with the interposition of a liner and/or sock. The socket transfers load between the person and the rest of the prosthesis in a manner that protects the health of residual limb tissues and allows control of the prosthetic limb. The socket is thus shaped (rectified) to properly interface with the anatomy and clinical presentation of each unique individual while also being lightweight and structurally sound to support their activities of daily living. For this reason, it is custom designed by certified prosthetists and custom fabricated through traditional processes, such as vacuum forming or thermoforming and lamination, or through new processes such as 3D printing.

Despite the socket's central role in comfort and function, no standards or common guidelines exist to test their structural strength, either in ultimate failure load or in fatigue durability. Without standardized test methods, the socket mechanical properties remain largely unknown.^1^ Consequently, it is not possible to complete a risk analysis or to evaluate the repeatability of the fabrication process and account for the influence of the operator, which might be significant in the traditional custom-fabrication method.^2^ Therefore, risk management tends to rely on the addition of reinforcement until safety is assured, essentially overfabricating the socket.

Overfabrication has several downsides. First, it is rather wasteful of material, potentially adding to fabrication time and cost, and contributing to environmental pollution. Second, being associated with increased weight and rigidity, it may contribute to heat retention, perspiration, and suspension issues, which in turn contribute to patient discomfort. Finally, this approach has higher risk of failure when experimenting with new materials and fabrication methods, for which training and clinical rules of thumb are not yet established. For example, in the case of emerging 3D printed or commercially available patient-adaptable or volume-adjustable sockets, a smaller knowledge base may exist regarding failure modes or fabrication principles needed to avoid structural failure.

The current regulatory framework is moving in the direction of requiring socket testing. For example, the current European Medical Device Regulation (MDR 2017/745) requires documenting the expected performance of custom-made medical devices, such as sockets, for strength and fatigue durability.^3^ In the United States, the Food and Drug Administration (FDA) is also considering aligning prosthetic device regulations with other similar devices and with the European MDR^4^ (Supplemental Appendix A, http://links.lww.com/POI/A139). The European MDR also provides a framework and terminology for describing the different categories of socket manufacture that we will use throughout this discussion paper. These include mass produced, adaptable, patient matched, and custom made.^3,5,6^ The definitions of these terms are reported in Supplemental Appendix B (http://links.lww.com/POI/A139).

In addition, the International Organization for Standardization (ISO) standards for prosthetic component testing use the term “sample” when describing testing of a single specimen (e.g., a prosthetic foot) and “batch” when describing testing of a collection of specimens (e.g., multiple prosthetic feet). For consistency with the standards, we use this terminology in the paper but recommend that consideration should be given to changing it so as to avoid confusion with the statistical use of the term “sample” which refers to a subset drawn from a particular population. The need for distinction in terminology will become apparent when considering the need for data drawn from human subjects to inform aspects of socket testing.

The existing standardized test and characterization methods for lower limb prosthetic components and systems, such as ISO 10328, ISO 16955, and ISO 22675, do not allow assessment of sockets to meet the abovementioned regulatory requirements. International Organization for Standardization 16955 and ISO 22675 do not include the use of prosthetic sockets during testing. ISO 10328 describes test setups that include a portion of the socket; however, they focus only on the distal end where external components typically attach, but none of the test setups adequately consider the socket as a whole^7^ (Supplemental Appendix C, http://links.lww.com/POI/A139).

Without a means to assess the structural strength and durability of prosthetic sockets, innovators who develop novel prosthetic socket designs or materials do not have the ability to verify structural integrity before first use on humans. A failure of the socket can result in falls and injury, and therefore, every effort should be made to perform due diligence with mechanical testing before human use of novel socket technologies. Unfortunately, at this time the absence of such a test method prevents this structural testing. The lack of standardization may also reinforce overfabrication, stifling innovation that would benefit prosthesis users.

Assessing the structural performance of socket fabrication through the introduction of socket guidelines will benefit patients, clinicians, payors, and developers of new socket technologies. Meeting guidelines would engender confidence in all stakeholders that a manufacturer's production process generates sockets that satisfy minimum structural requirements for the product's intended use. The optimization of socket design and production after testing is expected to lead to sockets lasting longer, necessitating fewer repairs and replacements, and potentially reducing time and costs. It would also potentially benefit the environment by decreasing material waste and prolonging the useful cycle of sockets.

To support these needs, an international collaboration of experts was formed in 2020 to provide guidance on prosthetic socket structural strength and durability testing. The “AOPA Socket Guidance Workgroup” is a multidisciplinary group of experts hosted by the American Orthotics and Prosthetics Association that includes representatives of the clinical community, the ISO/Technical Committee 168 (Prosthetics and Orthotics) Workgroup 3 (Testing), manufacturers of mass-produced and adaptable medical devices, commercial providers of prosthetic sockets and/or socket materials, and both academic and government researchers from the United States, United Kingdom, and Europe.

The central goal of this Workgroup is to provide the prosthetic community with evidence-based recommendations regarding socket structural testing methods to meet the emerging regulatory requirements and support innovators. Hence, the aim of this discussion paper was to

1. describe the current state of knowledge available in the literature regarding structural testing of transtibial prosthetic sockets,
2. identify the knowledge gaps in this field, and
3. provide recommendations for how to address them.

These recommendations should serve as a roadmap for stakeholders to take coordinated action in their respective field of interest.

## Methods

The Workgroup met monthly from January 2020 to review the available evidence, identify gaps in knowledge, and consider possible approaches for addressing the gaps. Relevant to discussions were the references of the FDA and MDR to external limb prostheses and in particular to custom devices (summarized in Supplemental Appendix A, http://links.lww.com/POI/A139) and ISO 10328 methodology (summarized in Supplemental Appendix C, http://links.lww.com/POI/A139). Definitions of the terms related to medical devices that are used throughout this discussion paper are reported in Supplemental Appendix B (http://links.lww.com/POI/A139). The Workgroup identified the methods of ISO 10328 as a potential foundation for future prosthetic socket test methodology due to its influence on socket tests reported in the literature to date. The Workgroup also identified the main 2 coordinate systems and alignment methods for lower limb prosthetic sockets available in the literature (summarized in Supplemental Appendix D, http://links.lww.com/POI/A139). This paper summarizes the recommendations of the Workgroup; individual contributions of the Workgroup members are reported in the Author Contribution paragraph. A draft of this paper was shared in October 2022 with ISO/Technical Committee 168 Workgroup 3 (Testing) who provided review and additional comments.

The findings of a recent systematic review^1^ along with other literature considered by the Workgroup are organized thematically according to 4 “key domains”:

1. Mock residual limb model shape and composition.
2. Prosthetic socket coordinate systems and alignment.
3. Components and requirements of test specimens.
4. Test conditions, loading parameters, and acceptance criteria.

For each key domain, we provide a brief introduction, a summary of the currently available evidence, identify gaps in the evidence, challenges associated with addressing these gaps, possible outcomes if more knowledge were available, and recommendations for how to fill the gaps. Specifically, we formulated requirements and target questions to address each gap and proposed one or more solution approaches based on the 3 categories shown in Table 1.

**Table 1.**
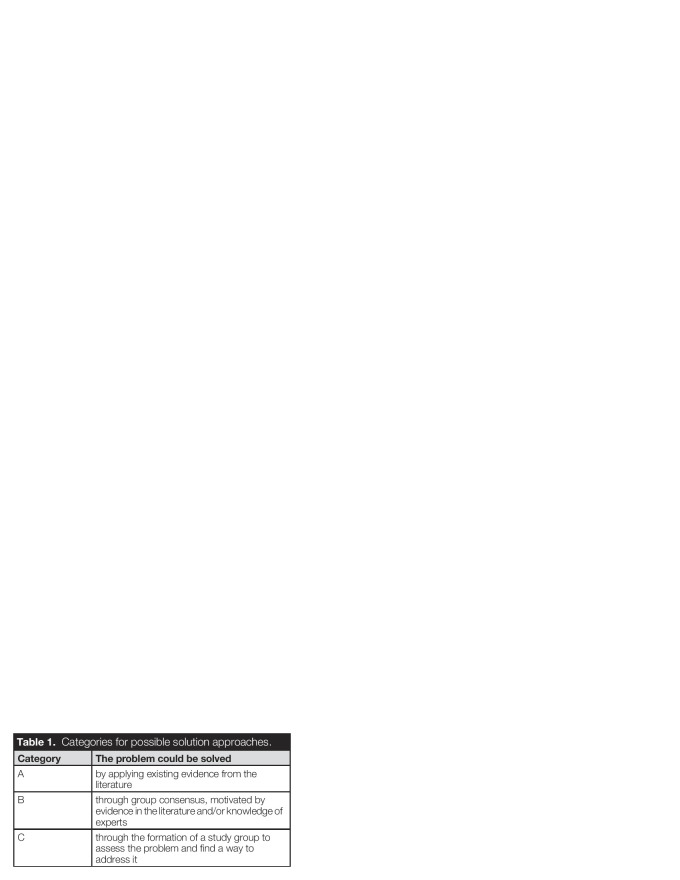
Categories for possible solution approaches.

Category	The problem could be solved
A	by applying existing evidence from the literature
B	through group consensus, motivated by evidence in the literature and/or knowledge of experts
C	through the formation of a study group to assess the problem and find a way to address it

## Results—Knowledge gaps and associated recommendations

### Key domain 1—Mock residual limb shape and composition

Socket structural tests will require a mock residual limb (also known in the literature as surrogate, analogue, or limb dummy). This is used to transfer the loads from the test machine to the socket during socket structural testing. Ideally, it should have appropriate surface and mechanical properties to provide load distribution patterns within the prosthetic socket that represent *in vivo* socket use. Moreover, it should be simple and repeatable to fabricate, durable under the test loading conditions, and have appropriate shelf life.

ISO 10328 does not describe the composition of a mock residual limb, but it contemplates its use when the complete prosthesis is tested. It suggests using a mock limb that is rigid, with a compliant or truncated (void) distal end and an embedded rigid rod for connection with the top lever arm of the test machine.^7^ The mock limb is not fully defined regarding the exact shape, the amount of distal end to be left void or filled with compliant material, and whether there is a compliant interface between the rigid limb model and the socket. The minimal guidance regarding mock limb construction provided by ISO 10328 has led to adoption in the literature of different mock limb designs for shape (truncated^8-15^ vs. full^16-19^), material composition (rigid^9-17^ vs. flexible^8^), interface (liner^9,15,16^ vs. no liner^8,10,12-14,17-19^), and limb shape (subject specific^15,18-20^ vs. generalized^8,10,12,13,16^).^1^ Moreover, only transtibial limb shapes have been described in published studies. As no sensitivity analysis exists to establish how these different factors affect the test results, the effect of one solution over another is unknown, which results in a lack of consensus among studies about a realistic yet most critical mock residual limb design.

Clarifying the construction of the mock limb model(s) based on field evidence will support standardization of the load distribution within the socket in testing and ensure that the entire socket receives relevant loading, rather than the distal end alone. Moreover, standardization of load application would allow test results to be comparable.

As shown in Table 2, 3 knowledge gaps were identified in this domain: the definition of a “reasonable” load distribution generated within the socket (Gap 1), the shape (Gap 2), and the composition (Gap 3) of the mock limb.

**Table 2.**
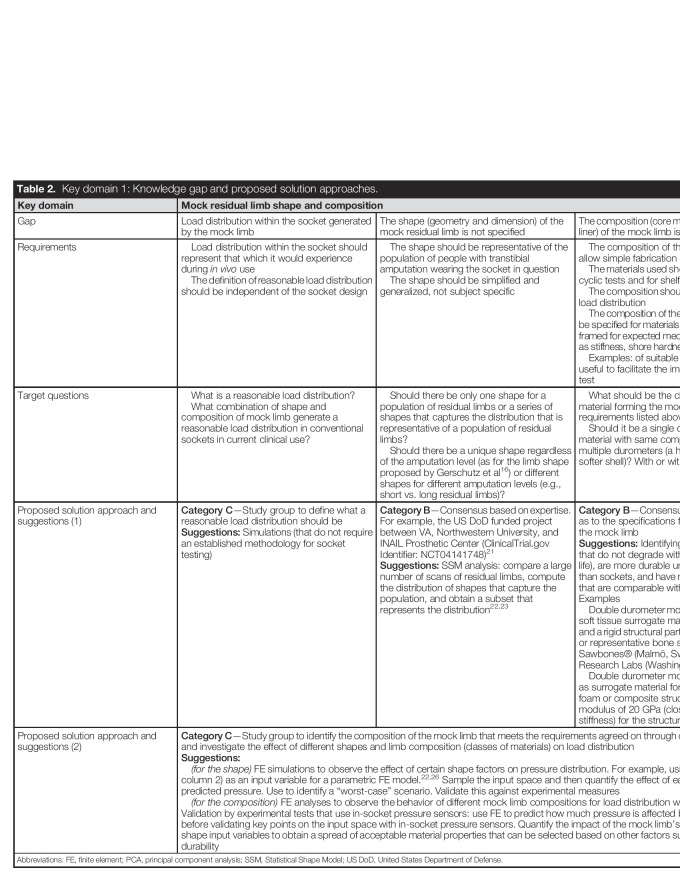
Key domain 1: Knowledge gap and proposed solution approaches.

Key domain	Mock residual limb shape and composition		
Gap	Load distribution within the socket generated by the mock limb	The shape (geometry and dimension) of the mock residual limb is not specified	The composition (core material(s) and possible liner) of the mock limb is not specified
Requirements	Load distribution within the socket should represent that which it would experience during *in vivo* use The definition of reasonable load distribution should be independent of the socket design	The shape should be representative of the population of people with transtibial amputation wearing the socket in question The shape should be simplified and generalized, not subject specific	The composition of the mock limb should allow simple fabrication The materials used should be durable under cyclic tests and for shelf life The composition should provide reasonable load distribution The composition of the mock limb should not be specified for materials but rather it should be framed for expected mechanical behavior, such as stiffness, shore hardness, etc. Examples: of suitable materials could be useful to facilitate the implementation of the test
Target questions	What is a reasonable load distribution? What combination of shape and composition of mock limb generate a reasonable load distribution in conventional sockets in current clinical use?	Should there be only one shape for a population of residual limbs or a series of shapes that captures the distribution that is representative of a population of residual limbs? Should there be a unique shape regardless of the amputation level (as for the limb shape proposed by Gerschutz et al^16^) or different shapes for different amputation levels (e.g., short vs. long residual limbs)?	What should be the characteristics of the material forming the mock limb (to meet the requirements listed above)? Should it be a single durometer (single material with same composition) or include multiple durometers (a harder core with a softer shell)? With or without a liner?
Proposed solution approach and suggestions (1)	**Category C**—Study group to define what a reasonable load distribution should be **Suggestions:** Simulations (that do not require an established methodology for socket testing)	**Category B**—Consensus based on expertise. For example, the US DoD funded project between VA, Northwestern University, and INAIL Prosthetic Center (ClinicalTrial.gov Identifier: NCT04141748)^21^ **Suggestions:** SSM analysis: compare a large number of scans of residual limbs, compute the distribution of shapes that capture the population, and obtain a subset that represents the distribution^22,23^	**Category B**—Consensus based on expertise as to the specifications for the composition of the mock limb **Suggestions:** Identifying classes of materials that do not degrade with time (sufficient shelf life), are more durable under cyclic condition than sockets, and have mechanical properties that are comparable with a human limb. Examples Double durometer mock limb, with a flexible soft tissue surrogate material for the bulk part and a rigid structural part, such as rigid pylon^24^ or representative bone surrogate such as Sawbones® (Malmö, Sweden)/Pacific Research Labs (Washington, DC)^25^ Double durometer mock limb with silicone as surrogate material for the bulk part and a foam or composite structure with a Young modulus of 20 GPa (close to the bone stiffness) for the structural part
Proposed solution approach and suggestions (2)	**Category C**—Study group to identify the composition of the mock limb that meets the requirements agreed on through consensus (column 1) and investigate the effect of different shapes and limb composition (classes of materials) on load distribution **Suggestions:** *(for the shape)* FE simulations to observe the effect of certain shape factors on pressure distribution. For example, using a PCA model (see column 2) as an input variable for a parametric FE model.^22,26^ Sample the input space and then quantify the effect of each shape variable on predicted pressure. Use to identify a “worst-case” scenario. Validate this against experimental measures *(for the composition)* FE analyses to observe the behavior of different mock limb compositions for load distribution within the socket. Validation by experimental tests that use in-socket pressure sensors: use FE to predict how much pressure is affected by mock limb material before validating key points on the input space with in-socket pressure sensors. Quantify the impact of the mock limb's material relative to its shape input variables to obtain a spread of acceptable material properties that can be selected based on other factors such as adherence and durability		

Abbreviations: FE, finite element; PCA, principal component analysis; SSM, Statistical Shape Model; US DoD, United States Department of Defense.

### Key domain 2—Prosthetic socket coordinate systems and alignment

The definition of a 3-dimensional reference system of orthogonal coordinates for each component and the complete test sample (i.e., specimen) is essential for the definition of any test configuration. Reference systems are necessary to establish the relative position and orientation of the components to be tested within the test frame. Traditional engineering verification methods place the test sample into the worst possible configuration for any test to ensure that under all anticipated conditions of normal use the product can withstand test loading. Therefore, it is advisable to define both a neutral (default) alignment and a worst alignment that puts the socket in the worst possible condition, making the test more conservative. This also requires identification of traumatic or abnormal use conditions that may need to be considered outside the scope of the tests.

ISO 10328 defines only one reference system for the complete prosthetic structure, referred to as the “o-f-u reference system.” It is based on the following reference points and axes: the knee joint center and centerline, the ankle joint center and centerline, and the foot longitudinal axis.^7^ However, this reference system is limited in that it cannot express the relative orientation between components, such as the alignment between the socket and the other distal elements. Moreover, it cannot be used when the socket is tested in isolation because it is defined using landmarks and axes of distal and proximal prosthetic components (i.e., ankle joint center, knee joint center, and foot long axis). In addition, the landmarks and axes on which this reference system is constructed are loosely defined in the standard.^7^

No other reference system has been defined in studies that performed socket testing^1^; almost all tend to use the ISO 10328 o-f-u reference system. Earlier literature about socket reference systems^27-31^ (from 1975 to 1999) provided a definition of the socket longitudinal axis and a method to locate it (Supplemental Appendix D, http://links.lww.com/POI/A139), and in 2020, Migliore et al^32^ proposed a method to identify the socket longitudinal axis through video analysis of the sagittal plane (Supplemental Appendix D, http://links.lww.com/POI/A139). However, it has not yet been reported whether the identification of the socket longitudinal axis is feasible in the context of socket testing.

ISO 10328 does not provide an established method of identifying alignment among components. Nevertheless, in case the alignment of the test sample (i.e., specimen) is adjustable, the standard prescribes setting it to a worst-case alignment. It then describes a worst-case alignment only for those cases in which the alignment cannot be defined by the manufacturer. In those cases, the worst-case alignment is defined as adjusting the sample (i.e., specimen) 90% of the distance from neutral to extreme alignment in the direction away from the load line to increase the effective lever arm.^7^ By comparison, the literature describes 3 modes of alignment: neutral, worst-case with pylon, and worst-case without pylon, referred to respectively as the “Current,” “Neo,” and “Gerschutz” alignment modes in the systematic review by Gariboldi et al.^1^ Unfortunately, the descriptions of each alignment mode are limited and mostly focused on the sagittal plane. In addition, as mentioned above, there is no clear definition of the socket reference system that is needed to achieve each alignment mode. In 1986, Zahedi et al^30^ proposed a set of 6 parameters to be specified to completely define the position and orientation of transtibial sockets relative to other prosthetic components. However, it is not clear whether the identification of these parameters can be implemented simply in the context of socket testing.

The nonuniform geometrical shape of typical prosthetic sockets has led to difficulties, ambiguities, and misunderstandings in specifying or attempting to measure alignment.^30^ The prosthesis–residual limb interface also acts as a pseudojoint with relative movements,^33^ contributing further to complexity of prescribing alignment. Without an established and unified reference system, it is not possible to define an alignment, to compare results, or to establish a repeatable testing method among different studies, within the same facility and across different facilities. Without an established method to align the components to be tested, it is not possible to quantify the bending and torsional moments acting on any single component, particularly on the socket. Therefore, it is not possible to classify a certain alignment mode as neutral or to reach a consensus on the worst-case condition.

As shown in Table 3, 3 knowledge gaps were identified in this domain: a unified definition of a socket reference system (Gap 1), of alignment (Gap 2), and of worst-case alignment (Gap 3).

**Table 3.**
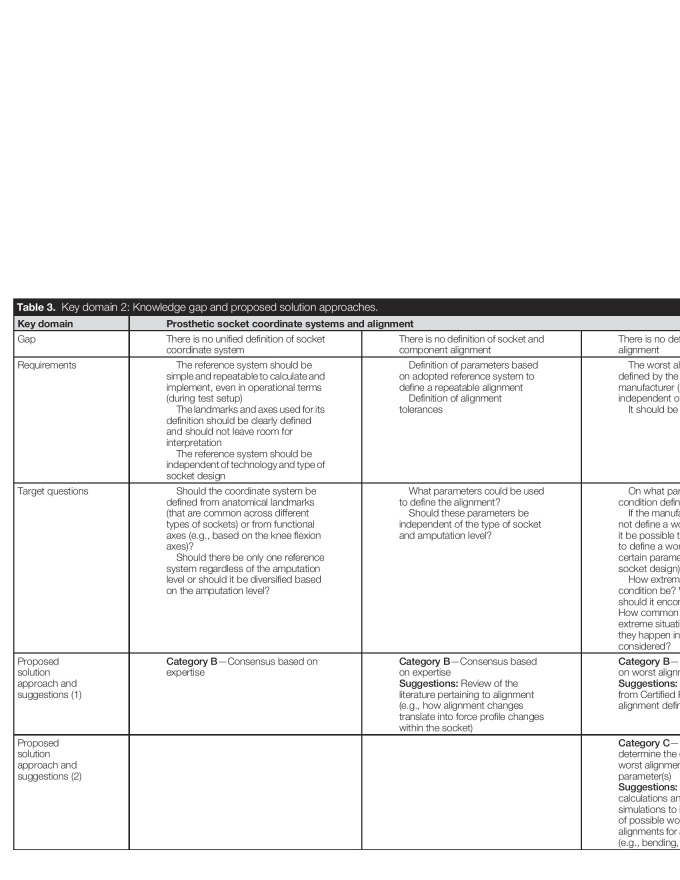
Key domain 2: Knowledge gap and proposed solution approaches.

Key domain	Prosthetic socket coordinate systems and alignment		
Gap	There is no unified definition of socket coordinate system	There is no definition of socket and component alignment	There is no definition of worst-case alignment
Requirements	The reference system should be simple and repeatable to calculate and implement, even in operational terms (during test setup) The landmarks and axes used for its definition should be clearly defined and should not leave room for interpretation The reference system should be independent of technology and type of socket design	Definition of parameters based on adopted reference system to define a repeatable alignment Definition of alignment tolerances	The worst alignment should be defined by the component manufacturer (so that it can be independent of socket design) It should be repeatable
Target questions	Should the coordinate system be defined from anatomical landmarks (that are common across different types of sockets) or from functional axes (e.g., based on the knee flexion axes)? Should there be only one reference system regardless of the amputation level or should it be diversified based on the amputation level?	What parameters could be used to define the alignment? Should these parameters be independent of the type of socket and amputation level?	On what parameter is the worst condition defined? If the manufacturer cannot or will not define a worst alignment, would it be possible to establish a method to define a worst alignment for a certain parameter (independently of socket design)? How extreme should the worst condition be? What use scenarios should it encompass and exclude? How common are the possible extreme situations? How often do they happen in the population that is considered?
Proposed solution approach and suggestions (1)	**Category B**—Consensus based on expertise	**Category B**—Consensus based on expertise **Suggestions:** Review of the literature pertaining to alignment (e.g., how alignment changes translate into force profile changes within the socket)	**Category B**—Consensus to agree on worst alignment **Suggestions:** Collect information from Certified Prosthetists on worst alignment definitions
Proposed solution approach and suggestions (2)			**Category C**—Study group to determine the effect of possible worst alignments for a certain parameter(s) **Suggestions:** Perform analytical calculations and numerical simulations to investigate the effect of possible worst-condition alignments for a certain parameter(s) (e.g., bending, torque, or axial load)

### Key domain 3—Components and requirements of test samples (i.e., specimens)

A test sample (i.e., specimen) is the element subjected to testing. It may coincide with the element that the user wishes to characterize, i.e., the prosthetic socket, or it may include additional elements that support the characterization of the main component (socket) such as the prosthetic pylon and/or foot. Other components that may be included may be elements that affect the structural integrity of the socket, such as valves, lanyard slots, and/or pylon mounting hardware. The omission of such components when appropriate could change the behavior of the socket and alter the test results.^1^

As specified above, ISO 10328 aims at characterizing mass-produced and adaptable prosthetic components, such as ankle-foot devices, knee joints, or knee locks, or the complete prosthetic structure. It does not encompass characterizing custom elements, such as the socket. The distal end of the socket may be included in the test sample (i.e., specimen) when testing the complete prosthetic structure but only if there is the need to provide remote support to demonstrate the strength of the mechanical connection between socket and distal components. The standard does not describe testing the entire socket inclusive of its proximal part, neither in isolation nor in combination with other elements.^7^

Although the available literature on socket testing^1^ mainly refers to ISO 10328, it does so with adaptations. All adaptations maintained the same test configurations and loads described in ISO 10328 for the principal structural tests, with the addition of a complete socket within the test sample (i.e., specimen). While most adaptations consisted of testing the socket together with the pylon, a few studies tested the socket in isolation.

As shown in Table 4, one knowledge gap was identified in this domain: define components of the test specimen (Gap 1).

**Table 4.**
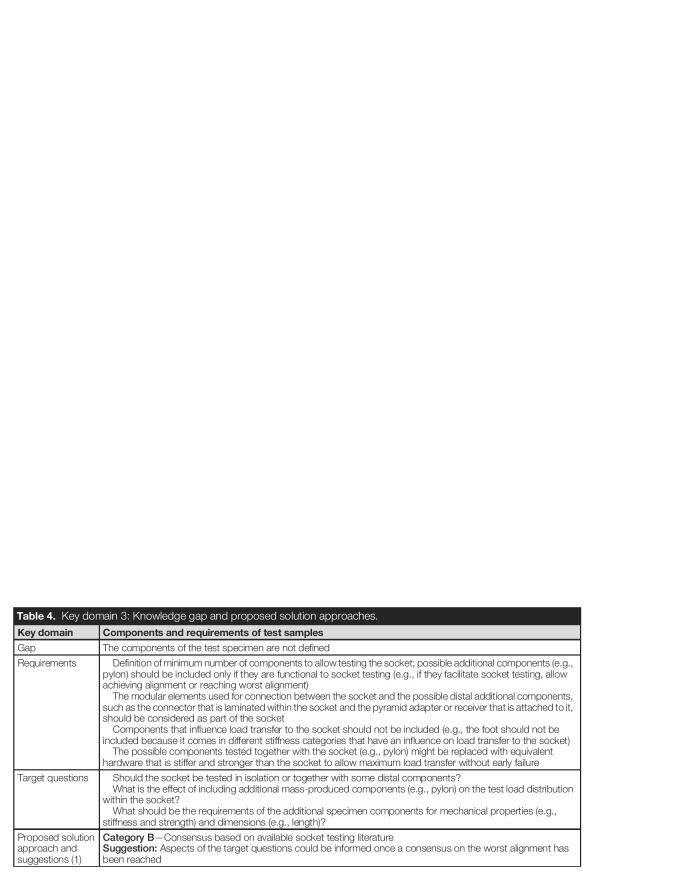
Key domain 3: Knowledge gap and proposed solution approaches.

Key domain	Components and requirements of test samples
Gap	The components of the test specimen are not defined
Requirements	Definition of minimum number of components to allow testing the socket; possible additional components (e.g., pylon) should be included only if they are functional to socket testing (e.g., if they facilitate socket testing, allow achieving alignment or reaching worst alignment) The modular elements used for connection between the socket and the possible distal additional components, such as the connector that is laminated within the socket and the pyramid adapter or receiver that is attached to it, should be considered as part of the socket Components that influence load transfer to the socket should not be included (e.g., the foot should not be included because it comes in different stiffness categories that have an influence on load transfer to the socket) The possible components tested together with the socket (e.g., pylon) might be replaced with equivalent hardware that is stiffer and stronger than the socket to allow maximum load transfer without early failure
Target questions	Should the socket be tested in isolation or together with some distal components? What is the effect of including additional mass-produced components (e.g., pylon) on the test load distribution within the socket? What should be the requirements of the additional specimen components for mechanical properties (e.g., stiffness and strength) and dimensions (e.g., length)?
Proposed solution approach and suggestions (1)	**Category B**—Consensus based on available socket testing literature **Suggestion:** Aspects of the target questions could be informed once a consensus on the worst alignment has been reached

### Key domain 4—Test conditions, loading parameters, and acceptance criteria

The test condition describes the set of parameters that allow replication of a certain situation of socket use that is considered representative, typical, critical, or extreme, whereas the loading condition describes how the load should be applied to the socket within that specific test condition (e.g., statically or cyclically). The outcome of the test should be compared with defined passing conditions or acceptance criteria.

ISO 10328 describes 2 types of tests: a principal test that recreates biplanar loading (flexion/extension and varus/valgus) and a separate torsion test (internal/external rotation). For each test type, it defines 2 test conditions (I and II) and 6 load levels (P3 to P8) for the complete prosthetic structure. Test conditions I and II allow replication of heel loading and forefoot loading, respectively, which are considered the most critical events within the stance phase. For each test condition, the load levels define the lever arms that are needed to recreate the conditions of heel or forefoot loading for individuals with different ranges of body weight, in association with locomotion characteristics, and the intended use of the device. The standard defines 6 loading levels for adults (from P3 to P8) based on locomotion data from people with amputation whose body weights range from 60 kg (P3) to 175 kg (P8). Moreover, the standard describes the loading parameters that are needed for static and cyclic tests (e.g., loading rate, loading frequency, procedures, etc) and the acceptance criteria of load and number of cycles for each loading level and condition.^7^ More details about the test conditions, loading levels, and acceptance criteria of ISO 10328 are available in Supplemental Appendix C (http://links.lww.com/POI/A139).

The test conditions and loading parameters of ISO 10328 have proven to be reliable for mass-produced and adaptable prosthetic components and complete lower limb prosthetic structures and have brought about improvements in product structural integrity, durability, and reliability based on the experience of manufacturers. However, the test conditions and loading parameters relevant to prosthetic sockets may be different than for distal structural components or for the complete structure. Adaptations of the ISO 10328 test conditions and loading parameters already described in the literature^1^ may be a useful starting point, but other socket-specific conditions should also be considered. Moreover, the relevance of the ISO 10328 test load vector and magnitude for generating appropriate knee moment remain unclear. The knee, being very close to the socket, makes an ideal reference location from which to derive the desired load application to the socket. It is also unclear whether biplanar loading (flexion/extension and varus/valgus) is sufficient with a separate test in torsion or whether all 3 planes of loading should be considered simultaneously.

As shown in Table 5, one knowledge gap was identified in this domain: define loading conditions for the socket (Gap 1).

**Table 5.**
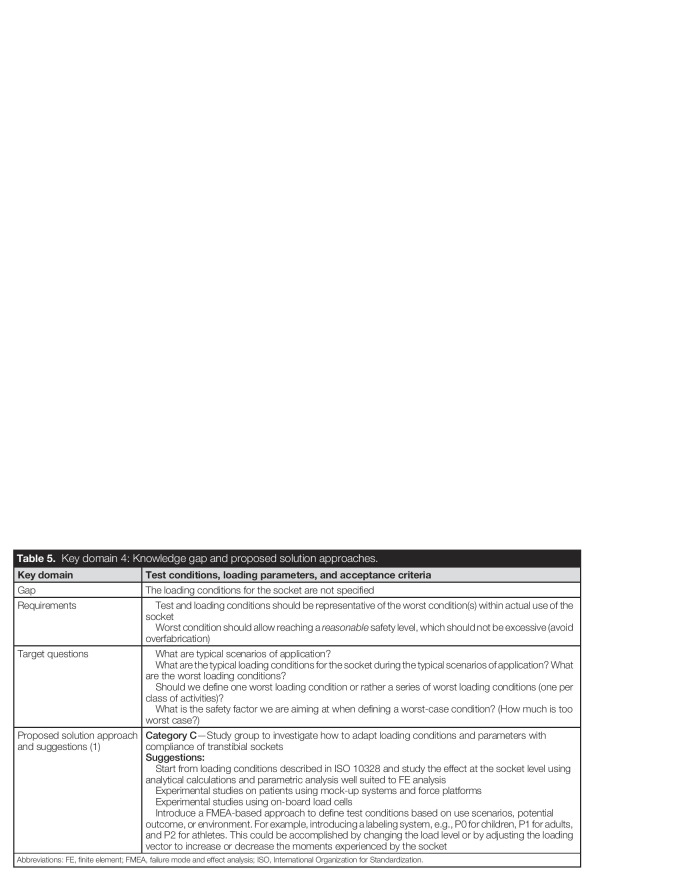
Key domain 4: Knowledge gap and proposed solution approaches.

Key domain	Test conditions, loading parameters, and acceptance criteria
Gap	The loading conditions for the socket are not specified
Requirements	Test and loading conditions should be representative of the worst condition(s) within actual use of the socket Worst condition should allow reaching a *reasonable* safety level, which should not be excessive (avoid overfabrication)
Target questions	What are typical scenarios of application? What are the typical loading conditions for the socket during the typical scenarios of application? What are the worst loading conditions? Should we define one worst loading condition or rather a series of worst loading conditions (one per class of activities)? What is the safety factor we are aiming at when defining a worst-case condition? (How much is too worst case?)
Proposed solution approach and suggestions (1)	**Category C**—Study group to investigate how to adapt loading conditions and parameters with compliance of transtibial sockets **Suggestions:** Start from loading conditions described in ISO 10328 and study the effect at the socket level using analytical calculations and parametric analysis well suited to FE analysis Experimental studies on patients using mock-up systems and force platforms Experimental studies using on-board load cells Introduce a FMEA-based approach to define test conditions based on use scenarios, potential outcome, or environment. For example, introducing a labeling system, e.g., P0 for children, P1 for adults, and P2 for athletes. This could be accomplished by changing the load level or by adjusting the loading vector to increase or decrease the moments experienced by the socket

Abbreviations: FE, finite element; FMEA, failure mode and effect analysis; ISO, International Organization for Standardization.

## Summary

The goal of this discussion paper was to provide the prosthetics community with evidence-based recommendations regarding the development of transtibial prosthetic socket structural testing methods to meet emerging regulatory requirements in the United States, United Kingdom, and Europe and support innovators in the field. The American Orthotics and Prosthetics Association Socket Guidance Workgroup identified knowledge gaps and solution approaches for 4 key domains necessary for defining such a test methodology:

1. Mock residual limb shape and composition.
2. Prosthetic socket coordinate system and alignment.
3. Components and requirements of test samples (i.e., specimens).
4. Test conditions, loading parameters, and acceptance criteria.

All 4 knowledge gaps were deemed to lack sufficient high-quality literature for resolving through literature review alone. The group concluded that many of the knowledge gaps within these domains may be resolved adequately by expert consensus combined with existing literature, but several knowledge gaps will require new research studies.

Proposed studies included

1. Defining load distribution applied to transtibial prosthetic sockets to reasonably represent use conditions (key domain 1, Table 2).
2. Assessing the effect of different mock limb shape and composition on the load distribution during testing within transtibial prosthetic sockets (key domain 1, Table 2).
3. Determining the mock limb composition that meets the requirements set forth by expert consensus through validation simulation or testing (key domain 1, Table 2).
4. Investigate the effect of possible worst-alignment conditions identified through consensus, using analytical and numerical calculations (key domain 2, Table 3).
5. Investigate how to adapt or replace ISO 10328 loading conditions and parameters to address the compliance of the mock limb within transtibial prosthetic sockets (key domain 4, Table 5).

The scope of this discussion paper was limited to transtibial sockets. Sockets for persons with transfemoral or knee disarticulation amputation will need to be considered separately. The authors are aware that knowledge about structural testing of upper limb sockets is also limited and represents an important gap that needs to be addressed. However, it entails very different use-case scenarios and injury risks. Quantifying the level of comfort related to the distribution of pressures at the limb–socket interface (with or without the interposition of a liner) is not considered part of a structural test method; therefore, this discussion paper does not consider clinical factors used in the rectification approach adopted for any particular socket design. The approach to testing was not specified and may include any type of analysis that allows structural assessment of prosthetic sockets (experimental or simulation). Ultimately, these methods should apply to any type of transtibial socket, regardless of the design, fabrication method, and material(s).

## Author contributions

All authors and Workgroup members were responsible for conceptualization. F.G. was responsible for writing-original draft. A.G.C., S.F., E.N., A.D., J.S., J.E., and S.Z. were responsible for writing-review & editing.

## Funding

Innovate UK (10014827; J.S., A.D.); Royal Academy of Engineering (EF1819\8\24; J.S.; RF/130; A.D.); The Alan Turing Institute, UK (UKRI EP/N510129/1; A.D.); VA-DoD Joint Incentive Funds (E.N.). For all other authors no sources of funding were declared.

## Declaration of conflicting interest

The authors disclosed no potential conflicts of interest with respect to the research, authorship, and/or publication of this article.

## Disclaimer

The opinions expressed in the article are the authors' and do not reflect the views of the Department of Veterans Affairs or the US Government.

## Acknowledgments

The AOPA Socket Guidance Workgroup kindly acknowledges the ISO/TC 168 (Prosthetics and Orthotics) WG3 (Testing) for their comments during the preparation of this manuscript. The Workgroup also acknowledges Susannah Engdahl for her commitment as Workgroup Manager, Jeffrey Erenstone for leading the Workgroup activities as Chair, and Joe McTernan for support to the Workgroup.

## Supplemental material

Supplemental material for this article is available in this article. Direct URL citation appears in the text and is provided in the HTML and PDF versions of this article on the journal's Web site (www.POIjournal.org).

## Appendix 1. AOPA Socket Guidance Workgroup

The AOPA Socket Guidance Workgroup includes Gary Berke, MS, CP, FAAOP, Department of Orthopaedic Surgery, Stanford University, Stanford, CA, USA; Berke Prosthetics & Orthotics, San Mateo, CA, USA; and Medical Creations, Denver, CO, USA. James Colvin, MSBME, WillowWood Global LLC, Mt. Sterling, OH, USA. Andrea Giovanni Cutti, MEng, PhD, CPO, INAIL Prosthetic Center, Vigorso di Budrio (BO), Italy. Drew Davis, BS, Minneapolis VA Health Care System, US Department of Veterans Affairs, Minneapolis, MN, USA. Alex Dickinson, MEng, PhD, Faculty of Engineering and Physical Science, University of Southampton, Southampton, United Kingdom. Susannah Engdahl, PhD, American Orthotic & Prosthetic Association, Alexandria, VA, USA. Jeffrey Erenstone, CPO, Performance Orthopedic Design, LLC, Lake Placid, NY, USA. Stefania Fatone, BPO(Hons), PhD, Department of Rehabilitation Medicine, University of Washington, Seattle, WA, USA. Francesca Gariboldi, MEng, Department of Industrial Engineering, University of Padua, Padua (PD), Italy. Samuel Hale, BSME, MSPO, Fillauer Companies Inc, Chattanooga, TN, USA. Charles King, CP, Arusha Control Inc, Cumberland, MD, USA, and O&P Virtual Library, Digital Resource Foundation for the Orthotics & Prosthetics Community, Gainesville, FL, USA. Glenn K. Klute, PhD, Department of Veterans Affairs, CliMB, Seattle, WA, USA, and Department of Mechanical Engineering, University of Washington, Seattle, WA, USA. William Layman, Department of Veterans Affairs, Hamilton, OH, USA. Giovanni Milandri, PhD, Department of Bioengineering, Imperial College London, London, United Kingdom. Eric Nickel, MS, Minneapolis VA Health Care System, US Department of Veterans Affairs, Minneapolis, MN, USA. Lonnie Nolt, MSME, WillowWood Global LLC, Mt. Sterling, OH, USA. Seth O'Brien, BA, CP, FAAOP(D), Otto Bock Patient Care, Phoenix, AZ, USA; American Academy of Orthotists & Prosthetists (AAOP), Falls Church, VA, USA. Nicola Petrone, MEng, PhD, Department of Industrial Engineering, University of Padua, Padua (PD), Italy. Jim Remley, BSME, Otto Bock Health Care, Salt Lake City, UT, USA. Joshua Steer, BEng, PhD, Radii Devices Ltd, Bristol, United Kingdom, and Faculty of Engineering & Physical Science, University of Southampton, Southampton, United Kingdom. Gregorio Teti, MEng, CPO, INAIL Prosthetic Center, Vigorso di Budrio (BO), Italy. Adan Vazquez, MEd, CP/LP, Department of Prosthetics and Orthotics, Alabama State University, Montgomery, AL, USA. Ashlie White, MA, Amputee Coalition, Washington, DC, USA. Brent Wright, BS, CPO, EastPoint Prosthetics and Orthotics Inc, Greater Raleigh Area, NC, USA. Lewis “Tres” Wright III, BS, CP, Medical Creations Inc, Denver, CO, USA. Shane R. Wurdeman, PhD, CP, FAAOP(D), Hanger Inc, Austin, TX, USA. Saeed Zahedi, PhD, FREng, Blatchford, Basingstoke, United Kingdom.
